# Mid-wavelength infrared avalanche photodetector with AlAsSb/GaSb superlattice

**DOI:** 10.1038/s41598-021-86566-8

**Published:** 2021-03-29

**Authors:** Jiakai Li, Arash Dehzangi, Gail Brown, Manijeh Razeghi

**Affiliations:** Center for Quantum Devices, Department of Electrical Engineering and Computer Science, Northwestern University, Evanston, IL 60208 USA

**Keywords:** Electrical and electronic engineering, Materials science, Nanoscience and technology, Optics and photonics

## Abstract

In this work, a mid-wavelength infrared separate absorption and multiplication avalanche photodiode (SAM-APD) with 100% cut-off wavelength of ~ 5.0 µm at 200 K grown by molecular beam epitaxy was demonstrated. The InAsSb-based SAM-APD device was designed to have electron dominated avalanche mechanism via the band structure engineered multi-quantum well structure based on AlAsSb/GaSb H-structure superlattice and InAsSb material in the multiplication region. The device exhibits a maximum multiplication gain of 29 at 200 K under -14.7 bias voltage. The maximum multiplication gain value for the MWIR SAM-APD increases from 29 at 200 K to 121 at 150 K. The electron and hole impact ionization coefficients were derived and the large difference between their value was observed. The carrier ionization ratio for the MWIR SAM-APD device was calculated to be ~ 0.097 at 200 K.

## Introduction

Mid-wavelength infrared (MWIR) photodetectors which can operate under the low flux conditions are of great interest for long-range military and astronomical applications^1,2^. In most of these applications there is a need to increase the capability of the system to detect light in a low photon flux situation. Therefore, gain-based devices such as heterojunction phototransistors (HPTs) and avalanche photodiodes (APDs) are used to achieve the necessary photoresponse when the incoming photon flux is low^3–5^. Compared with the HPTs, the APDs can amplify weak signals without the relatively more complicated HPT device structure^6^.

For MWIR APD devices, HgCdTe is the state-of-art material system and has been widely used in infrared APDs^7,8^. However, the HgCdTe-based APDs suffer from drawbacks such as material instability and low fabrication yields^9,10^. The emerging material system, antimony-based strained layer superlattices (SLS) have drawn lots of attention due to the advantages of high material uniformity, great bandgap tunability and Auger recombination suppression compared with HgCdTe detectors^11–14^. Recently, the MWIR APDs based on III–V superlattices have been demonstrated^15^. However, their performance, especially the excess noise factor, is limited due to the relatively small difference in the ionization rates for electrons and holes or because both electrons and holes are injected into the multiplication region. The SAM structure can be used to reduce excess noise factor and also enhance the multiplication noise gain through impact-ionization engineering^16,17^.

Due to the great band structure engineering flexibility, an antimonide-based SLS can be used as the barrier layer for a multi-quantum well (MQW) heterostructure when combined with an InAsSb well. This MQW enables using the antimony-based SLS to engineer a large difference in the ionization rates for electrons and holes by designing the band discontinuities between well and barrier to have a large difference between conduction band offset (*∆E*_*c*_) and valence band offset (*∆E*_*v*_). As demonstrated by McIntyre^18^, a large difference in the ionization rates for electrons and holes is essential for a low noise avalanche photodiode. Therefore, it is promising to use the MQW approach, with antimony-based SLS barriers, as the multiplication layer in the APDs. The separate absorption layer used in the SAM-APD is an InAsSb alloy chosen to cover the mid-infrared range at an operating temperature of 150 K.

The MWIR SAM-APD device was grown by molecular beam epitaxy with the multiplication layer consisting of an AlGaAsSb/InAs_0.9_Sb_0.1_ MQW. The AlGaAsSb barrier layer in the MQW structure was grown as an AlAs_0.1_Sb_0.9_/GaSb superlattice, This SLS is called an H-structure superlattice and can be used as an electron barrier layer in some type-II superlattice infrared photodetector designs^19^. For convenience, ‘AlGaAsSb’ will be used to refer to the AlAsSb/GaSb H-structure superlattice in the rest of discussion. The bandgap energy of the H-structure superlattice was calculated to be around 1 eV at 150 K by the empirical tight-binding method (ETBM) with sps* formalism which was modified from previous work^20,21^. The ETBM material parameter sets in the previous work were used^21^. The effective conduction band of the H-structure superlattice moves upward significantly due to the confinement of the electrons in the GaSb well by the AlAsSb barrier layers. The InAs_0.9_Sb_0.1_ bulk material was also used as the absorption layer for the mid-wavelength infrared detection at 150 K. During the growth, all the InAs_0.9_Sb_0.1_ layers in the device structure was grown as InAs-InSb binary-binary digital alloy^22^.

**Figure 1:**
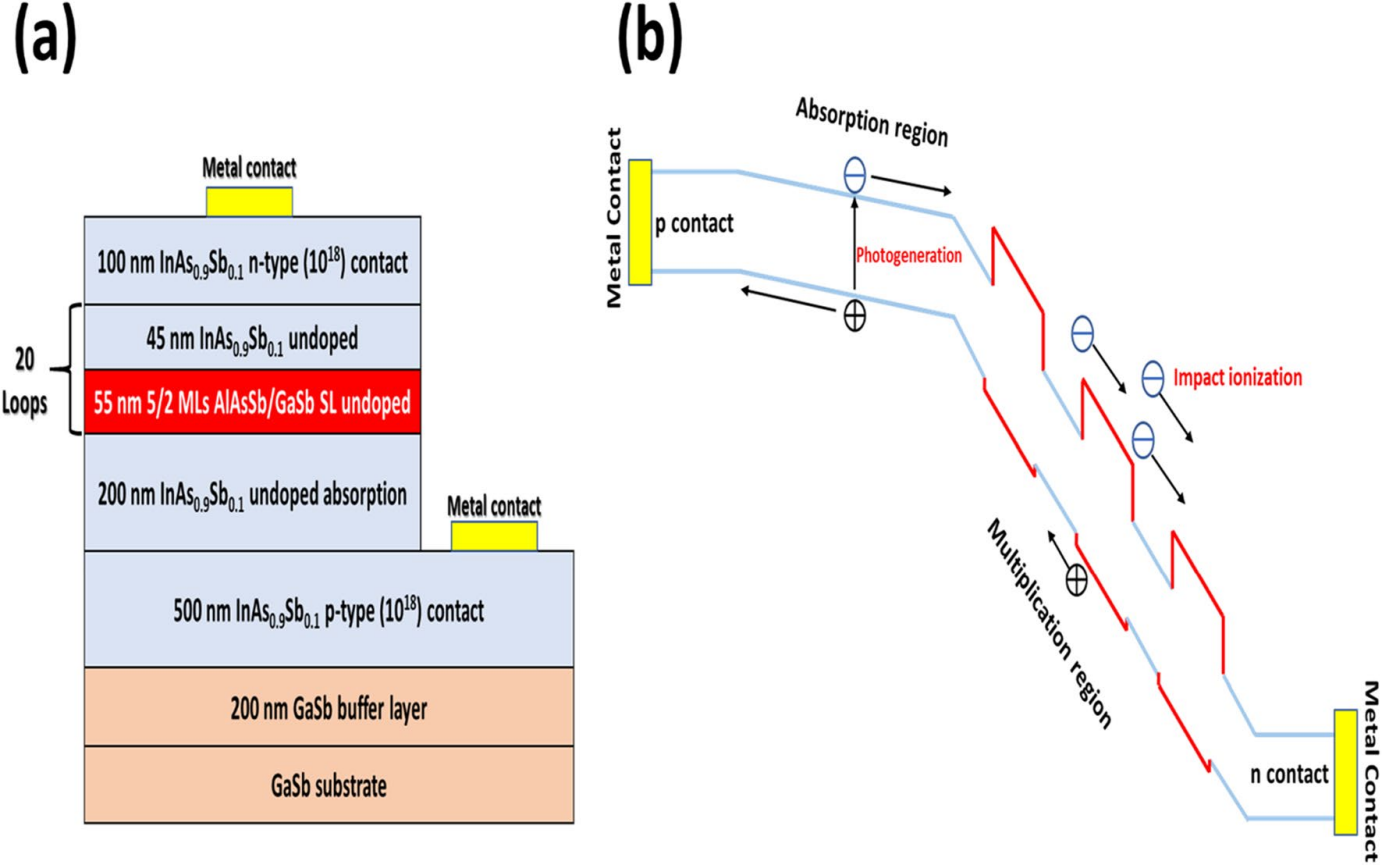
(**a**) Schematic of the MWIR SAM-APD structure. (**b**) Schematic band structure of the SAM-APD device under reverse bias.

The MWIR APD was grown on 2-inch Te-doped n-type (10^17^ cm^−3^) GaSb (100) substrate at 385 ℃ using an Intevac Modular Gen II molecular beam epitaxy (MBE) and its device structure is shown in Fig. 1(a). The 200 nm thick GaSb buffer layer and a 500 nm thick p-contact (10^18^ cm^−3^) InAs_0.9_Sb_0.1_ layer was grown. A 200 nm undoped InAs_0.9_Sb_0.1_ absorption layer was grown next. The InAsSb absorption layer was followed by a MQW structure with AlGaAsSb (AlAsSb/GaSb H-structure superlattice) as the barrier layer and InAs_0.9_Sb_0.1_ as the well layer. There are 20 loops of barrier and well layers in this MQW structure. The MQW structure was used as the multiplication layer, as shown in Fig. 1(b). To finish the device, a 100 nm top n-contact (10^18^ cm^−3^) InAs_0.9_Sb_0.1_ layer was grown. During growth, silicon and beryllium were used for n-type and p-type dopants, respectively. The schematic band diagram of the SAM-APD device under reverse bias is shown in Fig. 1(b).

**Figure 2:**
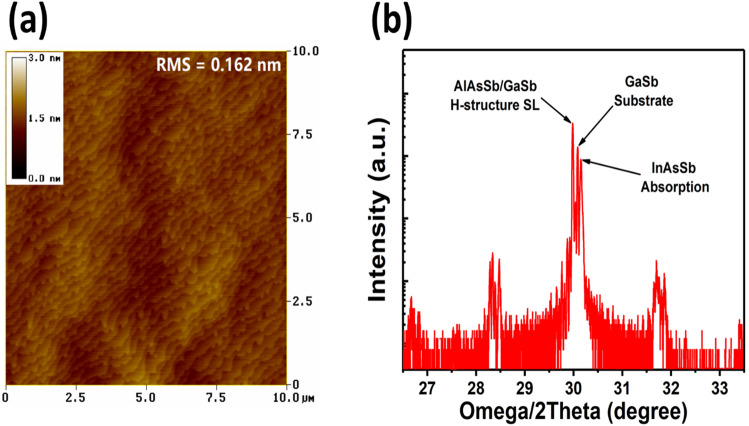
(**a**) The atomic force microscopy image of the grown MWIR APD sample over 10 μm × 10 μm area. (**b**) HR-XRD scan curve of the grown APD sample, where peaks of different regions are marked.

After the MBE growth, the material quality was assessed using atomic force microscopy (AFM) and high resolution X-ray diffraction (HR-XRD). As shown in Fig. 2(a), the sample surface exhibits clear atomic steps with a small RMS roughness of 0.162 nm over 10 μm × 10 μm area. Figure 2(b) shows the XRD scan curve of the MWIR APD sample, where the GaSb substrate, AlAsSb/GaSb H-structure SL and InAsSb layer peaks are marked. The mismatch between the AlAsSb/GaSb H-structure superlattice and the GaSb substrate is ~ 2500 ppm, while the InAsSb layer exhibits a negative mismatch of ~ − 1500 ppm. The higher-order satellite peaks in the XRD curve are not very strong and clear, which may be caused by the non-uniformity of the superlattice periodicity of AlAsSb/GaSb H-structure SL and can be further optimized by reducing the thickness of AlAsSb layers.

The conduction band offset (*∆E*_*c*_) is larger than the valence band offset (*∆E*_*v*_) between the barrier and well in the MQW structure according to the ETBM. As shown in Fig. 1(b), when a hot electron in the AlGaAsSb barrier of the MQW structure, accelerated by the applied bias voltage, enters a InAsSb well, it abruptly gains energy equal to the conduction band edge (*∆E*_*c*_). The net effect is that the electron sees a stronger electric field (increased by $\Delta$*E*_*c*_). Since the electron impact ionization rate in the InAsSb well layer, ${\alpha }_{InAsSb}^{{\prime}}$, increases exponentially with increasing electric field, a large increase in the electron impact ionization rate is expected. When the electron enters the next AlGaAsSb barrier, it sees a field decreased by *∆E*_*c*_ and thus a reduced electron impact ionization rate in the AlGaAsSb barrier layer ^23^. However, since ${\alpha }_{InAsSb}^{{\prime}}\gg {\alpha }_{AlGaAsSb}^{{\prime}}$, the exponential dependence on the threshold energy ensures that the average electron impact ionization rate $\stackrel{-}{\alpha }$:^24^

$$\stackrel{-}{\alpha }=\left({\alpha }_{InAsSb}^{{\prime}}{L}_{InAsSb}+{\alpha }_{AlGaAsSb}^{{\prime}}{L}_{AlGaAsSb}\right)/\left({L}_{InAsSb}+{L}_{AlGaAsSb}\right)$$

is largely increased (*L* denotes layer thicknesses). In contrast, the hole ionization rate β is not substantially changed by the MQW structure since the valence band discontinuity (*∆E*_*v*_) is much smaller. The holes can flow unhindered across the MQW multiplication layer. Therefore, the hole impact ionization rate β in the MQW structure is expected to have a similar value of β as in the bulk InAsSb material. In general, the difference between the electron impact ionization rate and the hole impact ionization rate can lead to a large reduction in the $\beta /\alpha$ ratio and lead to the pure or dominant electron-initiated multiplication mechanism in the MWIR APD.

The grown material was processed into a set of circular photodetectors using conventional photodetector processing^25,26^. An inductive couple plasma reactive ion etching (ICP-RIE) system followed by a citric acid-based isotropic wet etching solution of C_6_H_8_O_7_:H_2_O_2_:H_3_PO_4_∶H_2_O (5:2:1:20) was used during mesa etching to have smooth sidewalls. No anti-reflection (AR) coating was applied, and the devices were passivated for protection and insulation purposes by 600 nm SiO_2_ using plasma-enhanced chemical vapor deposition (PECVD). The test chip was then mounted onto a 68-pin leadless chip carrier (LCC) for electrical and optical characterization. The SAM-APD test chip was loaded into a Janis STVP-100 two chamber liquid helium cryostat station with controlled temperature ranging from 150 to 200 K.

**Figure 3:**
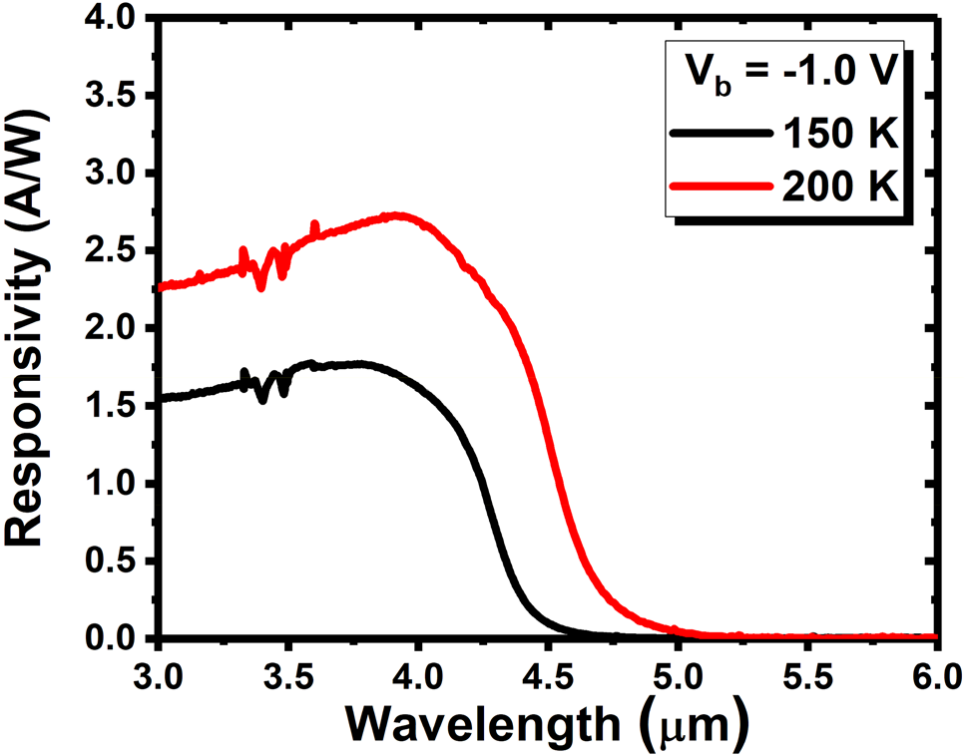
Responsivity spectra of the SAM-APD device under front-side illumination at 150 K (black) and 200 K (red) under − 1.0 V bias voltage.

The relative spectral response of the InAsSb-based MWIR SAM-APD was measured at both 150 K and 200 K under front-side illumination using a Bruker IFS 66v/S Fourier transform infrared spectrometer (FTIR). A calibrated blackbody source at 1000 ℃ was then used to calculate the absolute optical responsivity of the photodiodes^27^. The optical performance of the devices is shown in Fig. 3. At 150 K and 200 K, the responsivity for the SAM-APD device reaches a peak value of 1.71 A/W and 2.72 A/W at 3.9 µm under -1.0 V applied bias, respectively. The device exhibits a 100% cut-off wavelength of ~ 4.6 µm at 150 K and ~ 5.0 µm at 200 K.

**Figure 4:**
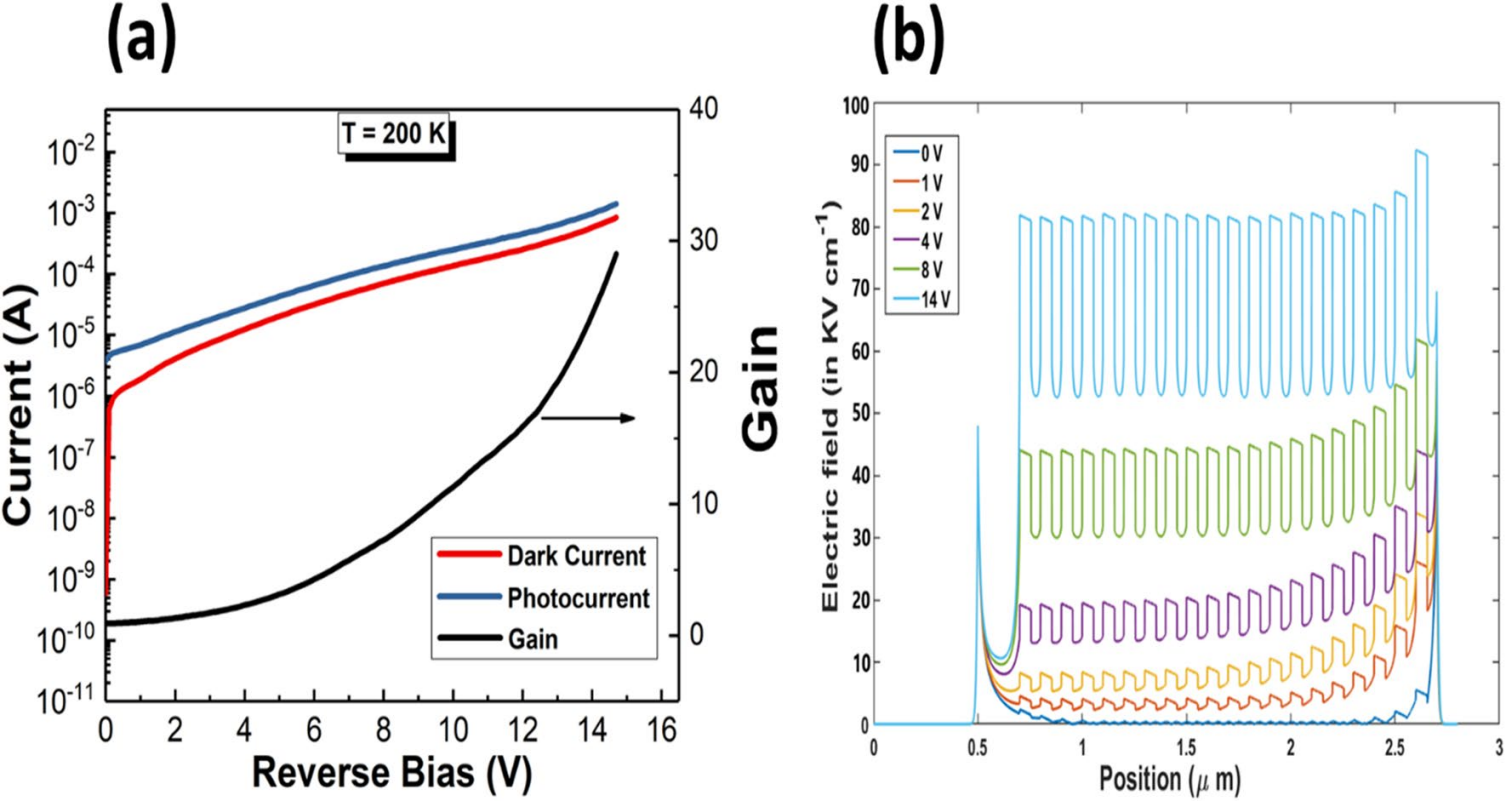
(**a**) Breakdown I-V characteristics and corresponding multiplication gain vs applied reverse bias at 200 K. Dark current and photocurrent are shown on the left axis; gain is shown on the right axis. (**b**) Simulated electric field profile under different applied reverse biases of SAM-APD structure.

Current–voltage (I–V) measurements of the SAM-APD sample were carried utilizing an Agilent 4156c semiconductor parameter analyzer. To study the temperature dependent gain characteristics, the measurement temperature for the MWIR InAsSb APDs was varied from 150 to 250 K. A 633 nm He–Ne laser with an incident power of 5.0 mW was used to measure the photocurrent and the gain of the APDs. The multiplication gain was calculated by normalizing the photocurrent, i.e. difference between light and dark currents, by the unity-gain photocurrent^28^. The typical breakdown I-V characteristics of a 200 × 200 µm^2^ MWIR InAsSb APD is shown in Fig. 4(a). A multiplication gain around 29 was achieved at a reverse bias voltage of − 14.7 V at 200 K. The unity-gain current at 200 K is 6.6 × 10^–6^ A at 0.9 V. As shown in Fig. 4(a), the gain exhibits an exponential increase as a function of the reverse bias. This demonstrates that the InAsSb-based SAM-APD has the expected exponential multiplication gain characteristic which confirms single carrier electron-dominated impact ionization in the avalanche regime, as seen in other MWIR APDs^29,30^. Figure 4(b) shows the simulated electric field intensity distribution across the SAM-APD device structure, especially the multiplication region using a 1D finite element model^31^. When the SAM-APD is under applied reverse bias, the electrons from the absorption region see the alternating strong and weak electric field in the MQW structure, as predicted theoretically in the previous part.

**Figure 5:**
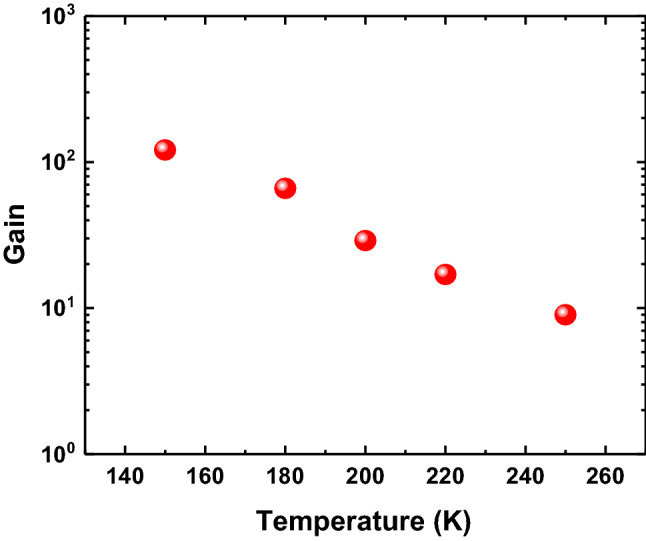
Temperature dependent gain characteristics of the InAsSb-based MWIR SAM-APD.

The relationship between the multiplication gain of the SAM-APD device and the temperature was also investigated as shown in Fig. 5. The temperature dependent gain characteristic exhibits the trend that the multiplication gain decreases continually from 121 to 10 while the temperature increases from 150 to 250 K. At 150 K, the maximum multiplication gain, around 121, for the MWIR SAM-APD device was achieved at -16.8 V bias voltage. The multiplication gain value for this device is larger than the previous reports for III-V materials based MWIR APDs^15^. The decrease of the multiplication gain at higher temperature is because the different scattering mechanisms, such as lattice scattering and impurity scattering, become stronger when the temperature increases, making a higher loss of kinetic energy for carriers via scattering. It is more difficult for the carriers to reach the impact ionization threshold energy at higher temperature. In addition, carrier-carrier scattering is an important factor in semiconductor materials where the impact ionization process is significant. The higher probability of carrier-carrier scattering at higher temperature also leads to the decrease of the multiplication gain for the APD device^32^.

**Figure 6:**
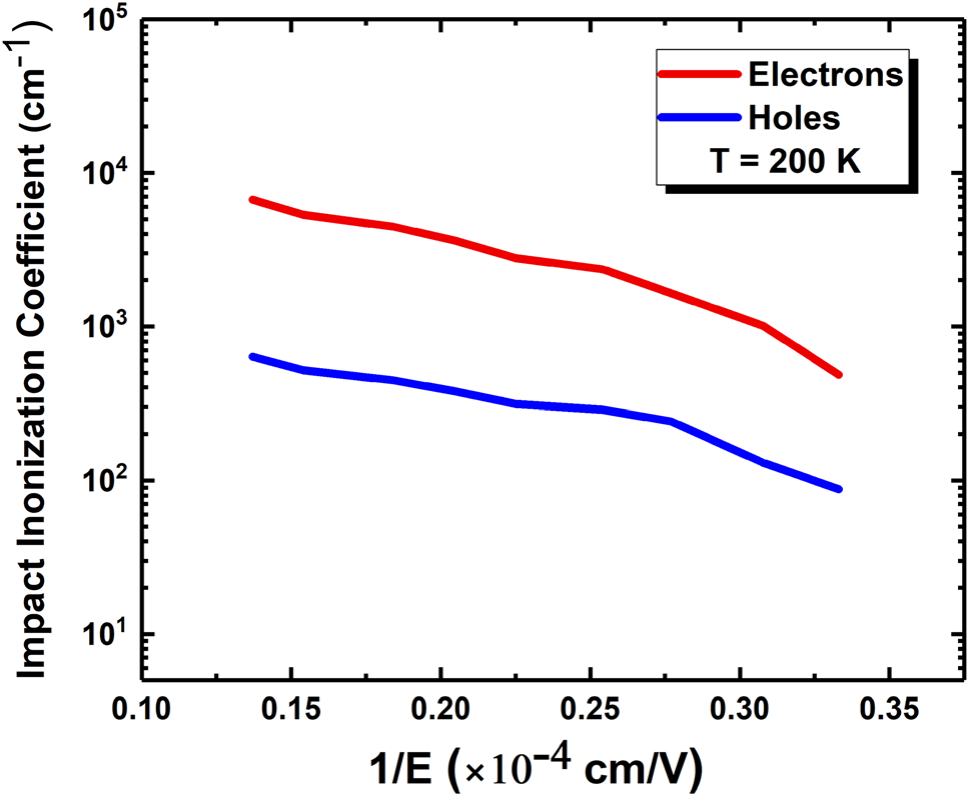
Electron and hole impact ionization coefficients for the MWIR SAM-APD vs inverse of electric field at 200 K.

Another APD device with the flipped structure (the p-type contact at the top and the n-contact at the bottom) was grown and processed under the same conditions to derive the electron and hole impact ionization coefficients, α and β from the experimental value of electron initiated avalanche gain ${M}_{e}$ and hole initiated avalanche gain ${M}_{h}$ by calculating the established formula^33^. The APD device with the flipped structure was illuminated from top p-type contact, where the carrier injection into the multiplication region is dominated by holes^34^. The derived electron and hole impact ionization coefficients are shown in Fig. 6, where the large difference between α and β is seen. The carrier ionization ratio, *k*, defined as the ratio of hole to electron impact ionization coefficient, for the MWIR SAM-APD was calculated to be ~ 0.097 at 200 K, which is smaller than the value of *k* (0.27) achieved in the previous report ^15^. This relatively small *k* value is largely due to the enhanced electron impact ionization in MQW multiplication region, which also agrees well with other simulation and experiment results of this effect in similar structures like superlattices in the previous works^24,35,36^. The small carrier ionization ratio is essential for achieving low excess noise as demonstrated by McIntyre^18^. The carrier ionization ratio is still larger than the *k* value (~ 0) achieved in InAs/GaSb superlattice based APD and can be further improved by tuning the band structure and loops of the MQW structure^29^.

In summary, the multi-quantum well structure using AlAsSb/GaSb H-structure superlattice was incorporated into an InAsSb-based SAM-APD device as the multiplication region. The SAM-APD device was grown on GaSb substrate by MBE and designed to have electron-dominated avalanche mechanism by engineering the electron impact ionization rate in the MQW structure. The device exhibits a 100% cut-off wavelength of ~ 4.6 µm at 150 K and reaches the peak responsivity of 1.71 A/W at 3.9 µm under -1.0 V applied bias. The multiplication gains of 29 were achieved for the SAM-APD device at 200 K, respectively. The carrier ionization ratio was calculated to be 0.097 at 200 K.

## Methods

### Growth

An Intevac Modular Gen II molecular beam epitaxy (MBE) equipped with group III SUMO cells and group V valved crackers was used to grow the SAM-APD structure on 2-inch Te-doped n-type (10^17^ cm^−3^) GaSb (100) substrate at 385 ℃. The growth rates for InAsSb, AlAsSb and GaSb are determined by the growth rate of group III elements and are about 0.5 ML/s, 0.35 ML/s and 0.75 ML/s, respectively. During growth, Silicon and Beryllium were used for n-type and p-type dopants, respectively.

### Fabrication

After growth, the wafer was fabricated into the two-contact mesa-isolated devices with the sizes varying from 40 × 40 μm^2^ to 300 × 300 μm^2^ using our standard single element photodiode fabrication steps. The photolithography was first used to create the pattern of mesa shapes and sizes on the photoresist above the sample. Then the combination of inductively coupled plasma-reactive ion etching (ICP-RIE) and wet etching was used to transfer the pattern of the photomask onto the sample to define the shape of the mesa. The wet etching right after ICP-RIE can remove the residue and smoothen the sidewall, which can reduce the surface leakage current. All the devices were cleaned thoroughly during processing to minimize the dark current. A second photolithography was used to define the top and bottom contact areas. Then the top and bottom metal contacts consisted of Ti/Au were deposited via electron beam metal evaporation. A SiO_2_ layer was deposited on the sample via plasma enhanced chemical vapor deposition (PECVD) to passivate the devices. The window to the metal contacts was opened by performing another photolithography and removing the SiO_2_ by reactive ion etching. The metal contacts for wire-bonding were then deposited.

### Device testing

The SAM-APD test chip was mounted onto a 68-pin leadless chip carrier (LCC) and then loaded into a Janis STVP-100 two chamber liquid helium cryostat station with controlled temperature ranging from 150 to 200 K. The relative spectral response of the InAsSb-based MWIR SAM-APD was measured at both 150 K and 200 K under front-side illumination using a Bruker IFS 66v/S Fourier transform infrared spectrometer (FTIR). A calibrated blackbody source at 1000 ℃ was then used to calculate the absolute optical responsivity of the photodiodes.

Current–voltage (I-V) measurements of the SAM-APD sample were carried utilizing an Agilent 4156c semiconductor parameter analyzer. A 633 nm He–Ne laser with an incident power of 5.0 mW was used to measure the photocurrent and the gain of the APDs at temperature range from 150 to 250 K.
